# No Evidence of Association between Common Autoimmunity *STAT4* and *IL23R* Risk Polymorphisms and Non-Anterior Uveitis

**DOI:** 10.1371/journal.pone.0072892

**Published:** 2013-11-29

**Authors:** María Carmen Cénit, Ana Márquez, Miguel Cordero-Coma, Marina Begoña Gorroño-Echebarría, Alejandro Fonollosa, Alfredo Adán, Agustín Martínez-Berriotxoa, David Díaz Valle, Esperanza Pato, Ricardo Blanco, Joaquín Cañal, Manuel Díaz-Llopis, José Luis García Serrano, Enrique de Ramón, María José del Rio, José Manuel Martín-Villa, Blanca Molins, Norberto Ortego-Centeno, Javier Martín

**Affiliations:** 1 Instituto de Parasitología y Biomedicina López-Neyra, IPBLN, CSIC, Granada, Spain; 2 Ophthalmology Department, Hospital de León, León, Spain; 3 Ophthalmology Department, Hospital Universitario Principe de Asturias, Alcalá de Henares, Spain; 4 Internal Medicine Department, Hospital de Cruces, Bilbao, Spain; 5 Ophthalmology Department, Hospital Clinic, Barcelona, Spain; 6 Ophthalmology Department, Hospital Clínico San Carlos, Madrid, Spain; 7 Rheumatology Department, Hospital Clínico San Carlos, Madrid, Spain; 8 Rheumatology Department, Hospital Marqués de Valdecilla, IFIMAV, Santander, Spain; 9 Ophthalmology Department, Hospital Marqués de Valdecilla, Santander, Spain; 10 Ophthalmology Department, Hospital Universitario La Fe, Valencia, Spain; 11 Ophthalmology Department, Hospital Clínico San Cecilio, Granada, Spain; 12 Internal Medicine Department, Hospital Carlos Haya, Málaga, Spain; 13 Ophthalmology Department, Hospital Carlos Haya, Málaga, Spain; 14 Immunology Department, Facultad de Medicina, Universidad Complutense de Madrid, Madrid, Spain; 15 Internal Medicine Department, Hospital Clínico San Cecilio, Granada, Spain; University of Birmingham, United Kingdom

## Abstract

**Objective:**

STAT4 and IL23R loci represent common susceptibility genetic factors in autoimmunity. We decided to investigate for the first time the possible role of different *STAT4*/*IL23R* autoimmune disease-associated polymorphisms on the susceptibility to develop non-anterior uveitis and its main clinical phenotypes.

**Methods:**

Four functional polymorphisms (rs3821236, rs7574865, rs7574070, and rs897200) located within *STAT4* gene as well as three independent polymorphisms (rs7517847, rs11209026, and rs1495965) located within *IL23R* were genotyped using TaqMan® allelic discrimination in a total of 206 patients with non-anterior uveitis and 1553 healthy controls from Spain.

**Results:**

No statistically significant differences were found when allele and genotype distributions were compared between non-anterior uveitis patients and controls for any *STAT4* (rs3821236: *P*=0.39, OR=1.12, CI 95%=0.87-1.43; rs7574865: *P*=0.59 OR=1.07, CI 95%=0.84-1.37; rs7574070: *P*=0.26, OR=0.89, CI 95%=0.72-1.10; rs897200: *P*=0.22, OR=0.88, CI 95%=0.71-1.08;) or *IL23R* polymorphisms (rs7517847: *P*=0.49, OR=1.08, CI 95%=0.87-1.33; rs11209026: *P*=0.26, OR=0.78, CI 95%=0.51-1.21; rs1495965: *P*=0.51, OR=0.93, CI 95%=0.76-1.15).

**Conclusion:**

Our results do not support a relevant role, similar to that described for other autoimmune diseases, of *IL23R* and *STAT4* polymorphisms in the non-anterior uveitis genetic predisposition. Further studies are needed to discard a possible weak effect of the studied variant.

## Introduction

Uveitis can be defined as any inflammation affecting the uveal tract, the middle vascular layer of the eye, although in the clinical practice this term includes any intraocular inflammatory process [1]. The prevalence of uveitis is estimated at 38 cases per 100,000 people, although it depends on the geographic area [1]. This condition is considered a major source of visual impairment as well as an important socio-economic problem, being the fourth cause of blindness on the worldwide [2]. There are different forms of uveitis, including either uveitis triggered by a wide range of exogenous (infectious and traumatism) or endogenous agents (non-infectious uveitis related with inflammatory or autoimmune processes) [3]. In addition, uveitis patients are frequently classified according to the anatomical location of the inflammation into anterior uveitis (AU), the most common form which represents 60% of cases, intermediate uveitis (IU) (5-13%), posterior uveitis (PU) (15%) and panuveitis or also called diffuse uveitis (PAN) (20%) [4]. 

Endogenous uveitis is an inflammatory response triggered by certain environmental factors in individuals with a particular genetic component and it is mainly mediated by immune system driven for a loss of tolerance against self antigens [3]. So far, certain HLA alleles have been strongly associated with the uveitis predisposition [5,6,7,8]. However, the effect of these alleles just explains a small part of the uveitis heritability and different studies have recently highlighted the implication of non-HLA genetic factors in the susceptibility to this condition [9,10,11]. 

There is a well established knowledge that most autoimmune diseases share a certain percentage of their genetic component, showing that some pathologies may be influenced by common pathways [12,13]. Genes encoding signal transducer and activator of transcription 4 (*STAT4*) and interleukin 23 receptor (*IL23R*) belong to the well established group of risk factors shared by different conditions that influence the breakdown of self-tolerance [13]. STAT4 and IL23R are strongly involved in the control of the immune system through of the development and perpetuation of Th17 immune responses [14], which display a dominant role in autoimmunity-associated inflammation [15]. 

Different studies have identified *IL23R* as a susceptibility factor associated to multiple inflammatory conditions [16,17,18]. In these studies several independent signals located within *IL23R* locus were suggested; however, only the R381Q (rs11209026) polymorphism, whose minor allele plays a protective role for several autoimmune disease, appear to have a functional involvement [19,20]. Additionally, the rs1495965 polymorphism has been reported as the stronger *IL23R* association with Behçet’s disease (BD), a systemic autoimmune disease involving uveitis, in a previous combined meta-analysis of two genome-wide association studies (GWASs) [21,22]. On the other hand, *STAT4* has been also identified as another shared susceptibility locus [23,24]. Interestingly, the presence of two *STAT4* independent functional genetic variants associated with systemic lupus erythematosus (SLE), both affecting the STAT4 levels, has been recently evidenced by fine mapping [25]. Furthermore, two functional polymorphisms located at *STAT4* gene have been recently implicated in BD susceptibility by GWASs [26,27]. 

Recent data have suggested that autoimmune uveitis also seems to share common genetic factors with other autoimmune disorders although these remain unknown yet [28]. Taking into account all of the above, we herein aimed to investigate whether the STAT4 and *IL23R* autoimmune disease-associated polymorphisms are involved in the genetic predisposition to autoimmune uveitis. 

## Methods

### Study population

#### Ethics statement

A subject’s written consent was obtained according to the declaration of Helsinki, and the design of the work was approved by the Ethics Committee of Granada (Spain). The Ethics Committees of the Hospital de León (León), Hospital Universitario Príncipe de Asturias (Alcalá de Henares), Hospital de Cruces (Bilbao), Hospital Clinic (Barcelona), Hospital Clínico San Carlos (Madrid), Hospital Marqués de Valdecilla (Santander), Hospital Universitario La Fe (Valencia), Hospital Clínico San Cecilio (Granada) and Hospital Carlos Haya (Málaga) also approved the study.

A total of 206 patients with endogenous non anterior uveitis, excluding the uveitis forms associated with systemic immune-mediated diseases except the Vogt-Koyanagi-Harada syndrome, and 1553 ethnically matched healthy controls, all of them of Caucasian origin, were included in the present study. The main clinical and demographic characteristics of uveitis patients included in the present study are described in Table **1**. The intraocular inflammation seen in patients included intermediate uveitis (24.8%), posterior uveitis (50.0%), and panuveitis (25.2%). 

**Table 1:** Clinical and demographic features of uveitis patients.

**General characteristics of uveitis patients**	**All patients n=206**
Female (%)	126 (61.2)
Age (mean ± SD)	47.3 ± 16.9
Intermediate Uveitis (%)	51 (24.8)
Posterior Uveitis (%)	103 (50.0)
Panuveitis (%)	52 (25.2)
Bilateral affection (%)	153 (74.3)
Vitritis (%)	135 (65.5)
Macular edema (%)	92 (44.7)
Retinal vasculitis (%)	87 (42.2)
Choroidal neovascularization (%)	16 (7.8)

### Genotyping

Genomic DNA was extracted from peripheral white blood cells and saliva following standard procedures. The single-nucleotide polymorphisms (SNPs) were genotyped using pre-designed TaqMan® allelic discrimination assays in a 7900HT Real-Time polymerase chain reaction (PCR) System from Applied Biosystems under conditions recommended by the manufacturer (Foster City, CA, USA). 

Since the aim of this study was to investigate whether different *STAT4*/*IL23R* autoimmune disease-associated polymorphisms were also implicated in the susceptibility to develop non-anterior uveitis, the *IL23R* and *STAT4* polymorphisms most robustly associated with autoimmunity were selected. Following this criterion, we studied the *IL23R* rs11209026 genetic variant, encoding the functional amino-acid change Arg381Gln [16,17,18,19,20], and two independent *STAT4* SNPs (rs3821236 and rs7574865), influencing levels of the protein [23,24,25]. Additionally, two *IL23R* polymorphisms were analyzed; rs7517847, strongly associated with Crohn’s disease and whose association seems to be independent on rs11209026 [16], and, rs1495965, previously associated with BD by GWASs [21,22]. On the other hand, two *STAT4* genetic variants, rs7574070 and rs897200, recently associated with BD, were included in the study [26,27]. Both polymorphisms seem to have functional consequences and are located in a different linkage disequilibrium block from the SNPs rs3821236 and rs7574865.

### Statistical Analysis

The overall statistical power of the analysis, according to Power Calculator for Genetic Studies 2006 software (http://www.sph.umich.edu/csg/abecasis/CaTS/), is shown in Table **S1**. The genotype, allele and carrier frequencies were compared between patients, subgroup of patients and controls applying χ^2^ test and/or Fisher’s exact test. The Linux software plink (v1.07) (http://pngu.mgh.harvard.edu/purcell/plink/) was used to perform 2 × 2 contingency tables and χ^2^ test and/or Fisher’s exact test when it was necessary. Odds ratios (OR) and 95% confidence intervals (CI) were obtained according to Woolf’s method. Hardy-Weinberg equilibrium (HWE) was tested for all SNPs at significance level=0.01. *P*-values below 0.05 were considered as statistically significant. 

Furthermore, the effect of the analyzed genetic variants was analyzed either in isolation or in allelic combination analysis. The allelic combination frequencies were estimated using plink (v1.07) and Haploview (V. 4.2).

Possible interactions between genes were evaluated following two different approaches: using logistic regression and by analyzing the distribution of genotypes for one SNP in cases conditioned by genotypes for the other SNP located in different gene.

## Results

Genotypic and allelic frequencies in cases and controls are shown in Table **2**. No statistically significant deviation from Hardy-Weinberg equilibrium (P≤0.01) was observed for all the studied SNPs in the control set and the frequencies of the analyzed SNPs were in agreement with the data of the HapMap project. Genotyping success rate was higher than 99% for all analyzed SNPs. In addition, randomly selected samples were genotyped twice to verify the genotyping accuracy and 99% of the genotypes were identical.

**Table 2:** Genotype and minor allele frequencies of *IL23R* and *STAT4* genetic variants in uveitis patients and healthy controls.

				**Genotype, N (%)**			**Allele test**	
**SNP**	**1/2**	**Subgroup (n)**	**1/1**	**1/2**	**2/2**	**MAF (%)**	P -value*	OR [CI 95%]**
***IL23R***								
rs7517847	G/T	Controls (n=1547)	267 (17.26)	732 (47.32)	548 (35.42)	40.92		
		Non anterior Uveitis (n=206)	41 (19.90)	94 (45.63)	71 (34.47)	42.72	0.49	1.08 (0.87-1.33)
rs11209026	A/G	Controls (n=1547)	8 (0.52)	211 (13.64)	1328 (85.84)	7.34		
		Non anterior Uveitis (n=206)	0 (0.00)	24 (11.65)	182 (88.35)	5.83	0.26	0.78 (0.51-1.21)
rs1495965	C/T	Controls (n=1547)	326 (21.07)	738 (47.71)	483 (31.22)	44.93		
		Non anterior Uveitis (n=206)	44 (21.36)	90 (43.69)	72 (34.95)	43.20	0.51	0.93 (0.76-1.15)
***STAT4***								
rs3821236	A/G	Controls (n=1547)	65 (4.20)	504 (32.58)	978 (63.22)	20.49		
		Non anterior Uveitis (n=206)	10 (4.85)	72 (34.95)	124 (60.19)	22.33	0.39	1.12 (0.87-1.43)
rs7574865	T/G	Controls (n=1547)	72 (4.65)	533 (34.45)	942 (60.89)	21.88		
		Non anterior Uveitis (n=206)	8 (3.88)	79 (38.35)	119 (57.77)	23.06	0.59	1.07 (0.84-1.37)
rs7574070	A/C	Controls (n=1547)	247 (15.97)	754 (48.74)	546 (35.29)	40.34		
		Non anterior Uveitis (n=203)	29 (14.29)	94 (46.31)	80 (39.41)	37.44	0.26	0.89 (0.72-1.10)
rs897200	T/C	Controls (n=1547)	277 (17.91)	759 (49.06)	511 (33.03)	42.44		
		Non anterior Uveitis (n=205)	33 (16.10)	95 (46.34)	77 (37.56)	39.27	0.22	0.88 (0.71-1.08)

*All P-values have been calculated for the allelic model; **Odds ratio for the minor allele.

As shown in Table **2**, when genotype, allele and carriers frequencies for the analyzed *STAT4* and *IL23R* SNPs were compared between non-anterior uveitis patients and controls, no association with the global disease susceptibility was observed for any of the analyzed polymorphisms. Similarly, when we compared the different subgroups of non-anterior uveitis patients stratified according to the different clinical features showed in Table **1** no statistically significant differences were detected (data not shown). 

On the other hand, the allelic combination analysis of the studied polymorphisms located within the *IL23R locu*s as well as within the *STAT4* gene did not provide further information (data not shown). 

Furthermore, since STAT4 and IL23R are molecules of the same pathway, we decided to study the possible interaction between the analyzed *IL23R* and *STAT4* genetic variants; however, no interaction between them was observed (data not shown). 

## Discussion

No large-scale genome-wide association studies in uveitis have been published to date, and only few genetic studies, focused mainly on anterior uveitis, have been conducted to identify the uveitis genetic component [9,10,11]. Indeed, only some HLA alleles have been strongly associated with uveitis predisposition [5,6,7,8], and genetic factors outside HLA region influencing uveitis susceptibility remain unknown. 

Autoimmune uveitis pathogenesis seems to be mainly mediated by a T cell-driven cellular immune response [29]. Uveitis condition was traditionally considered as a Th1 mediated trait [30]; however, cumulating knowledge suggests that IL-23, rather than IL-12, is necessary for experimental autoimmune uveitis (EAU) induction [31]. Indeed, Th17 cells, whose development in human is mainly induced in response to IL-23 and IL-1β [14], release interleukin-17A that exhibits potent proinflammatory properties in animal models of autoimmunity, including EAU [32]. In this IL-23/IL-17 axis, STAT4 is required for the signal transduction of IL-23 and, consequently is involved in the development of Th17 cells [33]. 

Considering the shared genetic component among the different immune-mediated diseases, including the autoimmune uveitis [12,13,28], and the key role that Th17 responses play in the uveitis pathogenesis, we speculated that *STAT4* and *IL23R*, two general autoimmunity *loci*, may contribute to the non-anterior uveitis susceptibility. 

However, our results evidenced no association of any analyzed *STAT4* and *IL23R* genetic variants with either the non-anterior uveitis or with the studied clinical subphenotypes. Regarding *IL23R*, the statistical power of our study to detect the described odds ratios in previous GWASs on different autoimmune diseases [16,34,35] was higher than 80%, considering the strong effect sizes observed in those studies for the analyzed *IL23R* variants (Table **3**). It could be speculated that the effect size of the analyzed *IL23R* polymorphisms was similar in non-anterior uveitis than in other autoimmune diseases. In this case, it is not likely that the lack of association was due to a type II error. Likewise, based on the statistical power of our study, an effect of the *STAT4* rs897200 and rs7574070 genetic variants similar to that described for BD [26,27] could be discarded in non-anterior uveitis (Table **3**). In relation to the *STAT4* rs3821236 and rs7574865 polymorphisms, there was a higher heterogeneity in the reported ORs [36,37,38,39,40] (Table **3**), with some autoimmune diseases showing considerably stronger signals than others (e.g. OR > 1.40 for SLE and 1.16 for RA). Therefore, no definitive conclusions on these *STAT4* variants can be drawn. 

**Table 3:** Statistical power of the present study accordingly with the described odds ratios for different autoimmune diseases in previous genome-wide association studies.

	**Disease**	OR^*^	**Statistical power**
***IL23R***			
rs7517847	IBD	0.62 [16]	0.99
rs11209026	CD	0.38 [34]	1.00
	UC	0.57 [35]	0.86
	AS	0.60 [45]	0.78
rs1495965	BD	1.35 [21]	0.81
***STAT4***			
rs3821236	SSc	1.30 [39]	0.53
	SLE	1.49 [38]	0.89
rs7574865	SSc	1.38 [36]	0.74
	SLE	1.49 [37]	0.91
	RA	1.16 [40]	0.21
rs7574070	BD	1.40 [26]/1.27 [27]	0.88/0.61
rs897200	BD	1.45 [41]	0.94

^*^ Odds ratio for the minor allele.

IBD, inflammatory bowel disease; CD, Crohn’s disease; UC, ulcerative colitis; AS, ankylosing spondylitis; BD, Behçet’s disease; SSc, systemic sclerosis; SLE, systemic lupus erythematosus; RA, rheumatoid arthritis.

On the other hand, associations of these *IL23R* and *STAT4* polymorphisms with systemic autoimmune diseases involving uveitis, such as Behçet’s disease, Vogt-Koyanagi-Harada disease or sarcoidosis, have been described in Asian populations [21,22,26,27,41,42,43,44] but not in Europeans. The allele frequencies of controls in our study are similar to those described in Asian populations. Therefore, the lack of association of these polymorphisms with non-anterior uveitis in the Spanish population may be due to a differential genetic background of this disease among ethnicities rather than differences in allele frequencies. Hence, these genes may not be uveitis risk factors in populations of European ancestry.

In conclusion, our data do not support a relevant role of *IL23R* and *STAT4* polymorphisms in the genetic susceptibility to develop non-anterior uveitis. As limitation, our study presents a moderate statistical power due to the low prevalence of endogenous non-anterior uveitis in Caucasian population. Hence, a possible weak effect of the studied variants in disease predisposition or phenotype expression may not be discarded and, hence, additional studies in larger independent cohorts would be desirable. 

## Supporting Information

Table S1 **Overall statistical power of the study for each analyzed IL23R and STAT4 genetic variant at the 5% significance level.**
(DOCX)
