# Supplementary material for: No Evidence of Association between Common Autoimmunity STAT4 and IL23R Risk Polymorphisms and Non-Anterior Uveitis
Source: PLoS One. 2013 Nov 29;8(11):e72892. doi: 10.1371/journal.pone.0072892 (PMC3843656; doi:10.1371/journal.pone.0072892)
Supplement: Table S1 — Overall statistical power of the study for each analyzed IL23R and STAT4 genetic variant at the 5% significance level. (DOCX) [file pone.0072892.s001.docx]

**SUPPORTING INFORMATION**

**Table S1.** Overall statistical power of the study for each analyzed *IL23R* and *STAT4* genetic variant at the 5% significance level.

|  | **OR=1.1** | **OR=1.2** | **OR=1.3** | **OR=1.4** | **OR=1.5** |
| --- | --- | --- | --- | --- | --- |
| ***IL23R*** |  |  |  |  |  |
| rs7517847 | 0.14 | 0.40 | 0.70 | 0.89 | 0.97 |
| rs11209026 | 0.07 | 0.14 | 0.25 | 0.39 | 0.55 |
| rs1495965 | 0.15 | 0.41 | 0.70 | 0.89 | 0.97 |
| ***STAT4*** |  |  |  |  |  |
| rs3821236 | 0.11 | 0.29 | 0.53 | 0.76 | 0.90 |
| rs7574865 | 0.11 | 0.29 | 0.55 | 0.77 | 0.91 |
| rs7574070 | 0.14 | 0.40 | 0.69 | 0.88 | 0.97 |
| rs897200 | 0.15 | 0.41 | 0.70 | 0.89 | 0.97 |

OR, odds ratio
